# Case Report: Defect repair post-resection of cervical tracheal granular cell tumor by cervical anterior banded myofascial flap: A case study and literature review

**DOI:** 10.3389/fonc.2023.1016232

**Published:** 2023-02-03

**Authors:** Zhu Liu, Zhendong Li

**Affiliations:** Department of Head and Neck Tumor Surgery, Cancer Hospital of Dalian University of Technology, Cancer Hospital of China Medical University, Liaoning Cancer Hospital & Institute, Shenyang, Liaoning, China

**Keywords:** granular cell tumor, tumor of the trachea, fascia tissue flap, tracheal membrane, tracheal reconstruction

## Abstract

**Objective:**

A case of cervical tracheal granular cell tumor (CTGCT) is reported together with a discussion on the clinical manifestation, diagnosis, and treatment of CTGCT. Additional cases of tumors in the tracheal membrane are also discussed. A simple and viable tracheal reconstruction method was proposed. The research design involves a case report and literature review.

**Methods:**

Twenty-four case reports on cervical GCT with complete clinical data were identified, with a specific focus on cases involving surgical treatment of tumors in the cervical tracheal membrane.

**Results:**

Twenty-eight reports of GCT in the cervical trachea and six reports on cervical tracheal membrane tumors were identified. The clinical data of a middle-aged Asian woman with a cervical GCT was also discussed.

**Conclusion:**

Cervical GCT is a rare disease, and tracheal resection is a reasonable treatment for cervical tracheal GCT. The proposed procedure is a simple and feasible method for reconstruction of the cervical tracheal membrane defect using a double-pedicled banded myofascial flap.

## Introduction

Granular cell tumors (GCTs) were first described by Abrikossoff in 1926 (1); the name derives from the presence of large numbers in eosinophilic granules in the tumor cell cytoplasm. GCT was initially considered a myogenic tumor and was described by Horn and Stout et al. (2) as a granular cell myoblastoma. Current pathological studies indicate that it is derived from neuronal tissue or Schwann cells (3, 4). It occurs most commonly in patients between the ages of 30 and 50 years old with a higher incidence in females than in males (5). Most of the tumors are benign, and only 1−2% are malignant (6, 7). GCT in the respiratory system accounts for 2−6% of the overall cases; most respiratory-associated GCTs occur in the larynx and tracheal GCT is rare. The treatment of cervical tracheal GCT includes the use of simple endoscopic forceps combined with laser, electrosurgery, and argon ion-assisted resection, together with open surgery (8). Here, the case and treatment of a middle-aged Asian woman with a cervical GCT is presented, together with a summary of the clinical data of 26 cases of cervical tracheal GCT and an analysis of the clinical manifestations, diagnosis, and treatment of the disease. This study focuses on the surgical methods used for treating cervical tracheal membrane tumors, and introduces a simple and feasible method for tracheal membrane reconstruction.

## Case report

**Figure 1:**
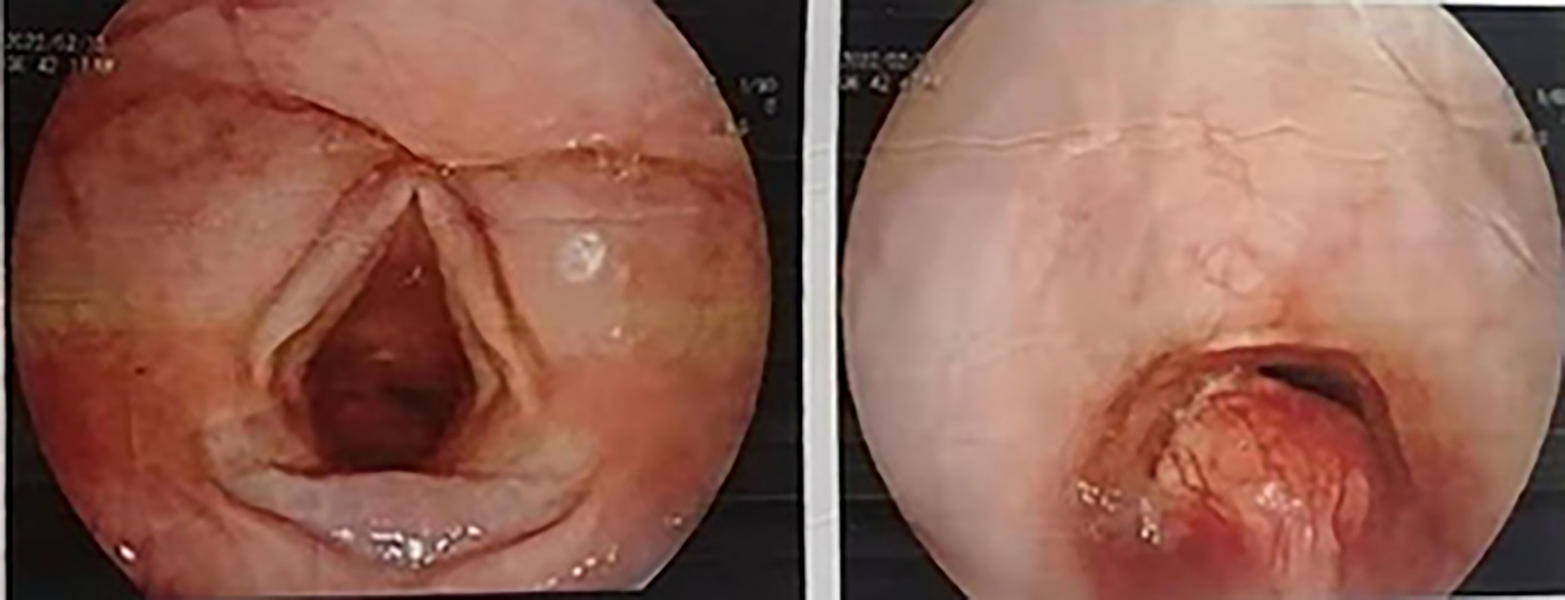
Bronchoscopic image; the scope positioned just cranial to the vocal cords shows the presence of a polypoid lesion within the infraglottic airway.

A 45-year-old Asian woman presented with a primary complaint of active dyspnea with irritant dry cough for two months. Bronchoscopy showed a bulging mass 2 cm below the voice box, which obstructed 80% of the lumen (**Figure 1**). The tumor was located in the submucosa, and biopsy specimens could not be obtained. There was no laryngeal vocalization, while the inspiratory and expiratory times were slightly prolonged, no swollen lymph nodes were found on the neck, and the breath sounds in both lungs were normal. No other abnormalities were observed. The patient was admitted to the hospital with clinical grade II dyspnea and moderate irritant airway spasm. The preoperative examination was completed within three days of admission. No biopsy was performed as the patient was unable to tolerate bronchoscopy. The assessment indicated no contraindications to surgery, and surgery was performed on the fourth day after admission. Prophylactic antibiotics were given half an hour before surgery, and the operation lasted for two hours. Postoperatively, the patient was admitted to the observation room in the ward. She had stable vital signs and did not experience respiratory distress, hoarseness, or hypocalcemia. Additionally, she was given continued cephalic antibiotics, phlegm, and intravenous nutrition treatment for a week. No local or intrapulmonary infection was observed. Hence, the patient was discharged after bronchoscopy on the ninth postoperative day.

**Figure 2:**
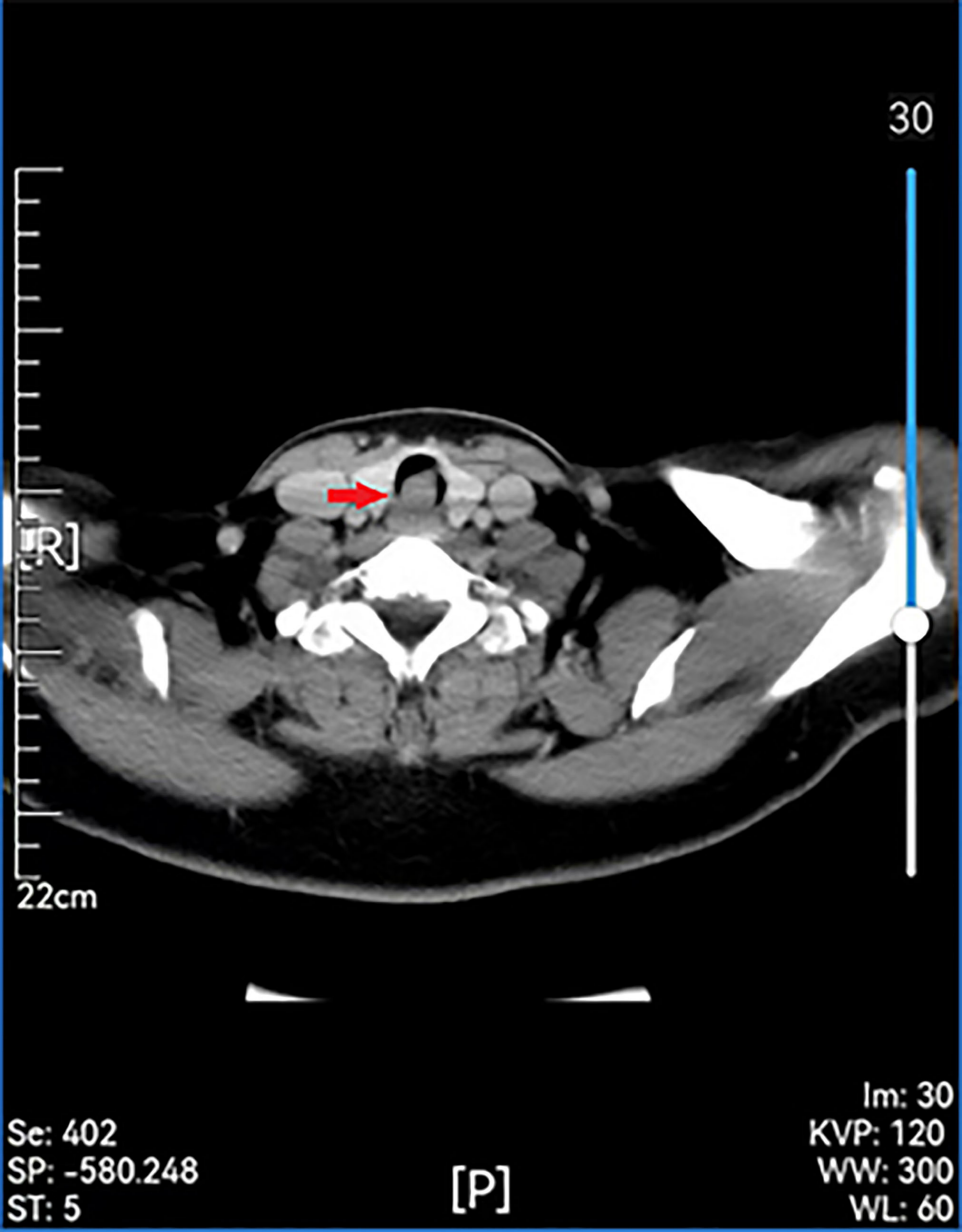
Preoperative enhanced CT scan showing a remodeled area of cervical with posterior wall erosion (red arrow).

**Figure 3:**
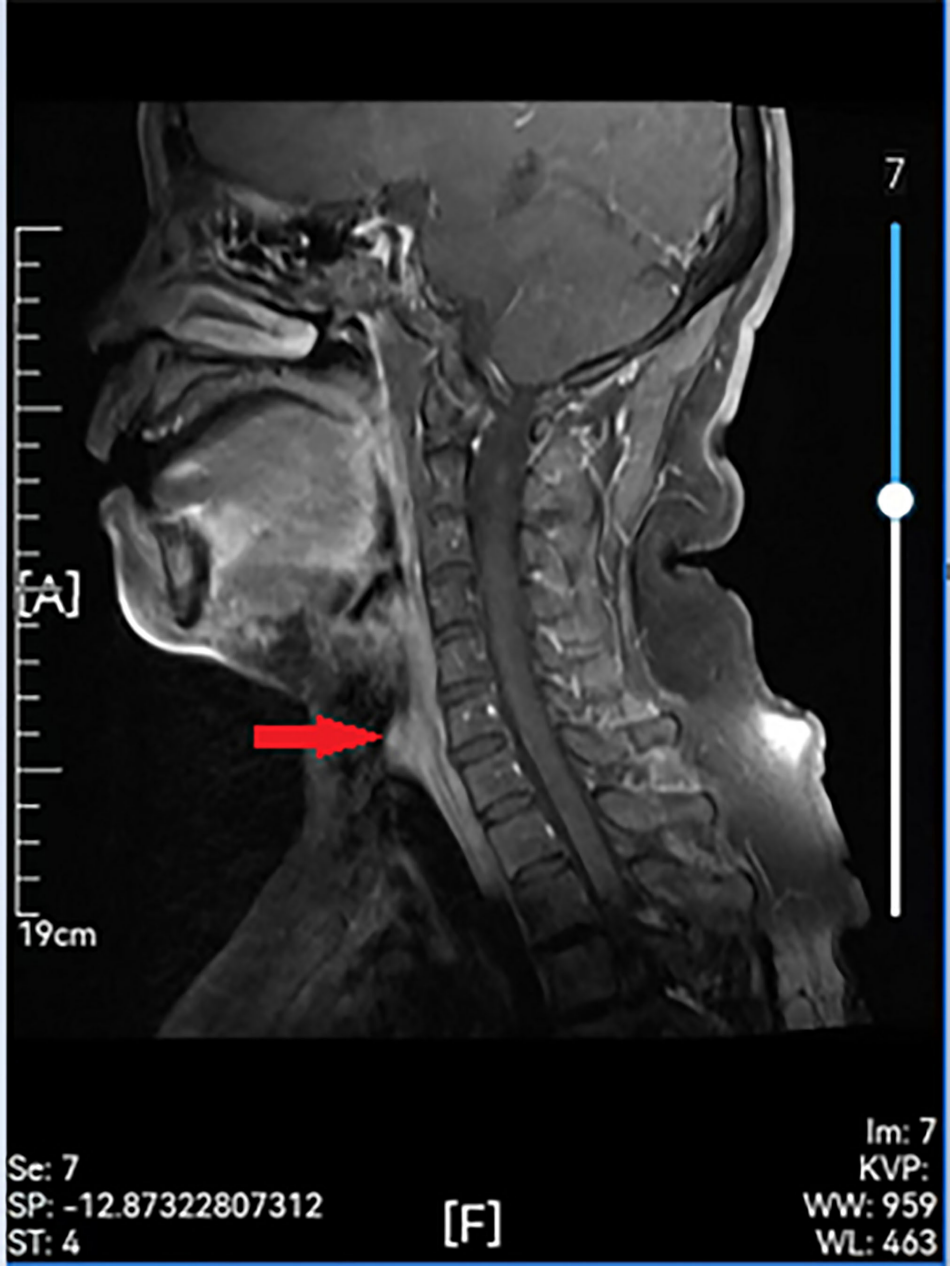
Preoperative enhanced MR showing a remodeled area of cervical with posterior wall erosion (red arrow).

Examinations showed that hematuria and stool routine were normal, coagulation routine was normal, and ECG was within the normal range. Enhanced computed tomography (CT) of the larynx and trachea revealed that the boundary between the tumor and the anterior wall of the esophagus was not clear, and the larger layer range was approximately 17 mm × 16 mm. This was apparent even after enhancement. The CT value of the plain scan was about 64 HU, and that of the enhanced scan was about 85 HU in the venous phase. Cervical posterior tracheal nodules were observed, and their nature was determined through microscopic examination (**Figure 2**). Enhanced MR in the neck revealed a nodular shadow in the cervical trachea of about 16 mm × 15 mm, and visible enhancement revealed long T1 and long T2 signals (**Figure 3**). Electronic duodenoscopy revealed no abnormality.

**Figure 4:**
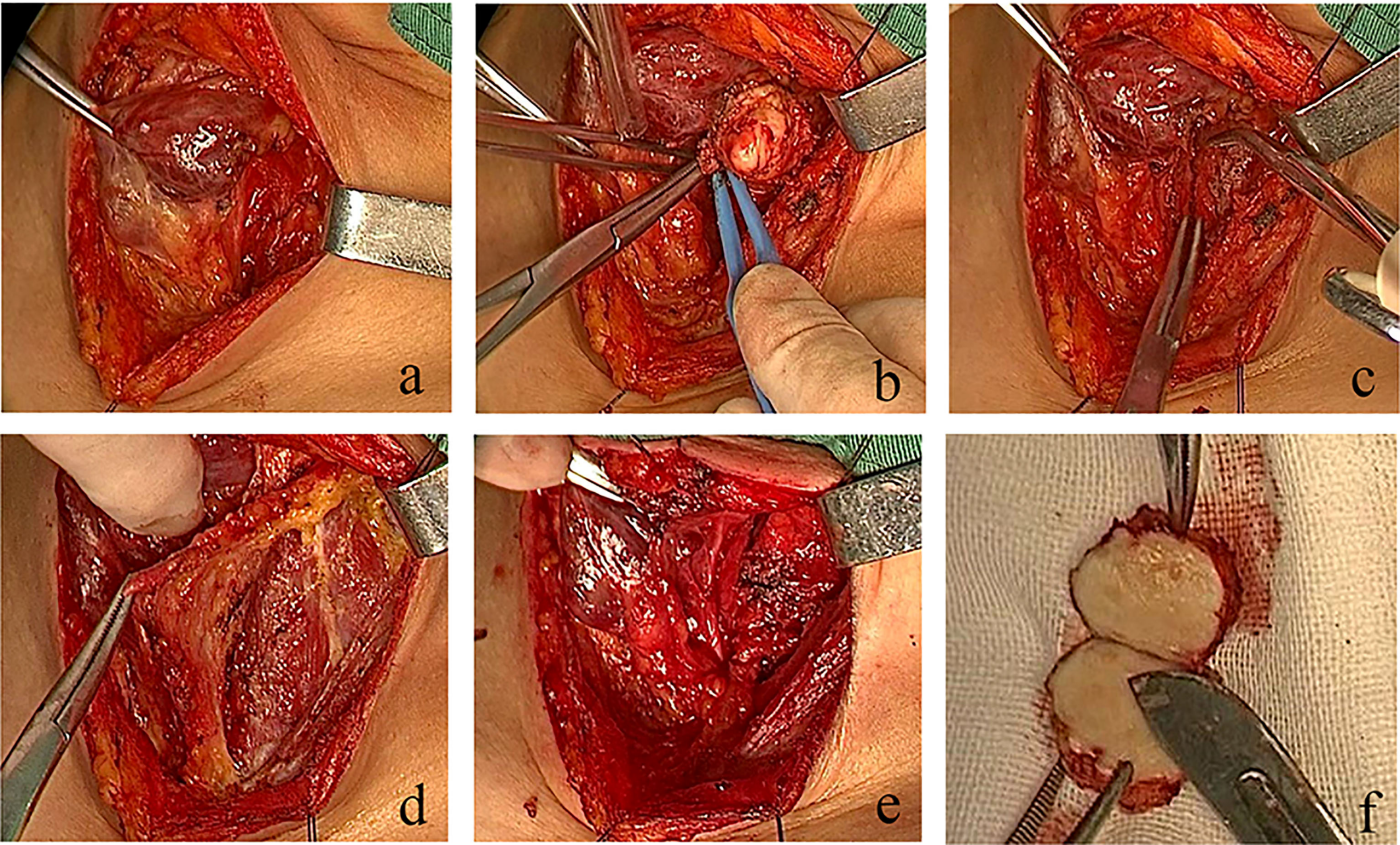
Real-time video of the operation process. In the case presented in this study, the defect was repaired by suturing the two lateral edges of the fascial tissue flap with the end of second to fourth tracheal rings and soft tissue in opposite positions. **(A)** Lateral freeing of the left thyroid gland, protection of the superior and inferior parathyroid glands, and the recurrent laryngeal nerve to expose the tumor. **(B)** Complete resection of the tracheal membrane tumor. **(C)** Tracheal membrane defect. **(D)** Preparation of a double-tipped banded myofascial flap. **(E)** No damage to the thyroid gland and recurrent laryngeal nerve after repair. **(F)** Specimen presentation.

**Figure 5:**
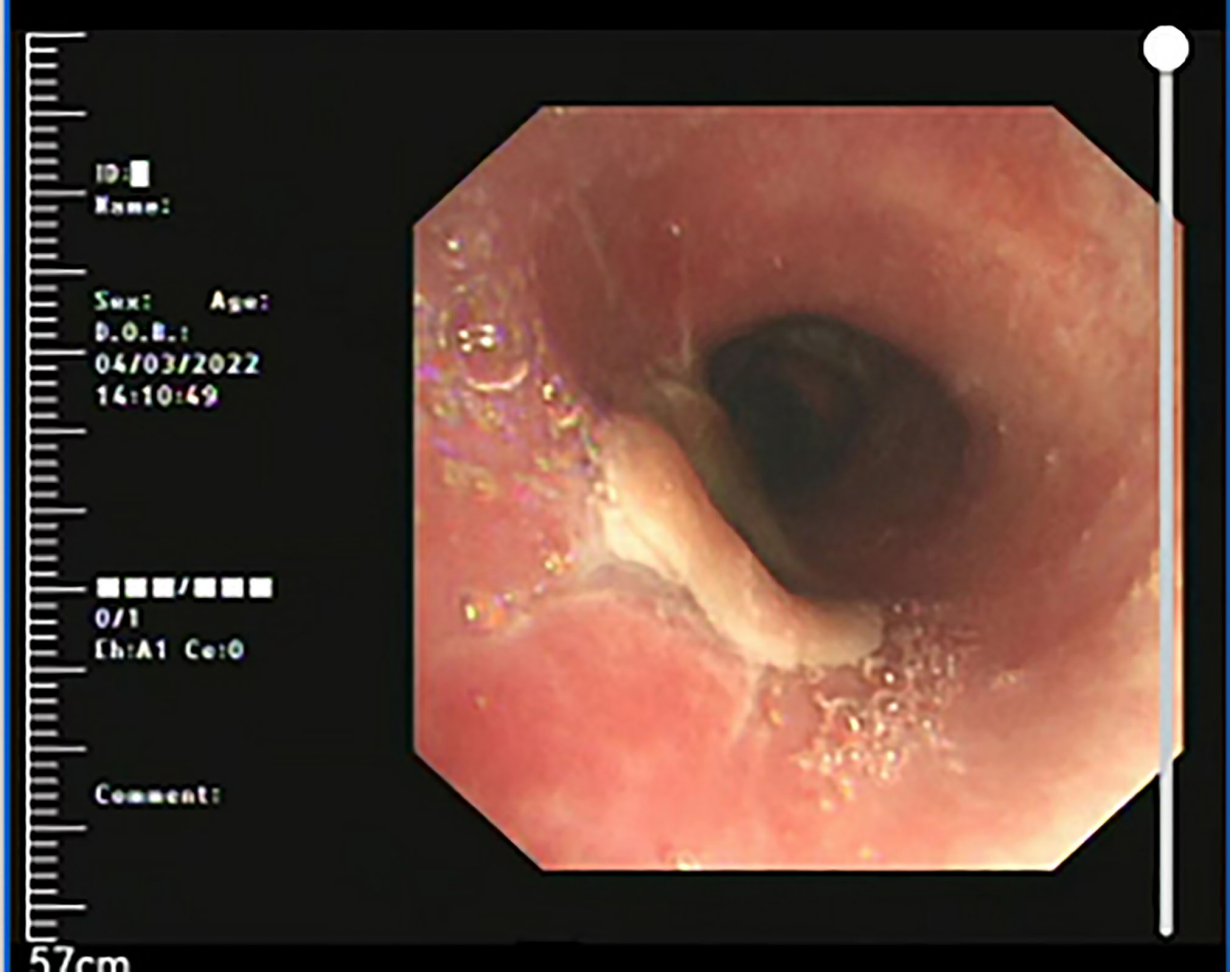
Three months after treatment, reexamination of bronchoscope showed a smooth surface of reconstruction site, with no tracheostenosis.

The tumor was exposed and removed after anesthesia induction, oral endotracheal intubation and mechanical ventilation, followed by the adjustment of the position of the endotracheal tube for tracheal defect repair. The tumor appeared as a smooth round-like shape in the tracheal cavity, located in the posterior membrane of the trachea. There was no stenosis in the trachea wall. The trachea cannula could pass through the tumor due to the elasticity of the posterior membrane. The patient was in the supine position. After intravenous anesthesia induction, a No. 6.5 reinforced endotracheal tube was used, and the cuff was 2 cm below the tumor, with cuff inflation. A low-collar incision in the neck was created. The left thyroid gland was exposed, and tissue separation was performed upward along the outer edge of the left thyroid gland. The middle thyroid vein and inferior thyroid artery were clamped and cut off under direct vision. The inferior thyroid pole was dissociated and exposed to protect the recurrent laryngeal nerve, and the lower parathyroid gland was retained *in situ*. The tissues along the surface of the recurrent laryngeal nerve were separated to the entrance point, with complete freeing of the lateral thyroid and retention of the upper parathyroid gland *in situ*, followed by the complete dissociation of the lower pole. The tissue block was then turned to the opposite side, and sharp dissection was performed on the surface of the trachea wall. The gland and isthmus were dissociated to the opposite lobe, and the recurrent laryngeal nerve was completely exposed and retained *in situ*. The tumor, with a length of approximately 2.0 cm, was located in the middle of the posterior of the second to fourth tracheal rings. The tumor was resected by an inter-tracheoesophageal approach, resulting in a tracheal membrane defect of approximately 3.0 cm × 2.0 cm after resection with a safe incision margin of 0.3 cm. Intraoperative pathological examination suggested the presence of a mesenchymal tumor. The fascia was cut on both sides of the left banded muscle to reach the muscle surface, and the full-thickness rectangular fascial flap of about 5×3×0.5 cm^3^ on the muscle surface was lifted as a whole from top to bottom and from outside to inside to keep the upper and lower pedicle blood supplies. The double pedicle was moved to the defect site without torsion, and a 3-0 absorbable suture was used for suturing with a needle distance of about 0.5 cm. The defect was repaired by suturing the two lateral edges of the fascial tissue flap with the end of the second to fourth tracheal rings and soft tissue in opposite positions (**Figure 4**). The tissue was sutured from both ends to the center without knotting the retained suture. When sewing and knotting the middle part, the endotracheal tube was lifted intermittently with suspended ventilation. All stitches were knotted after the suture. A temporary tracheal cutaneous fistula was performed by transverse incision of 1 cm at the sixth to seventh tracheal rings without an endotracheal cannula. Postoperative nasal and oral respiration was smooth, antibiotics were given for one week, no postoperative complications were observed, and the tracheocutaneous fistula was closed. Bronchoscopy examination on the ninth day after surgery showed a slight bulge 2-6 cm below the glottis, behind the second to fourth tracheal rings, with white moss attached (**Figure 5**). No recurrence has been observed in postoperative follow-up examinations up to the present date.

**Figure 6:**
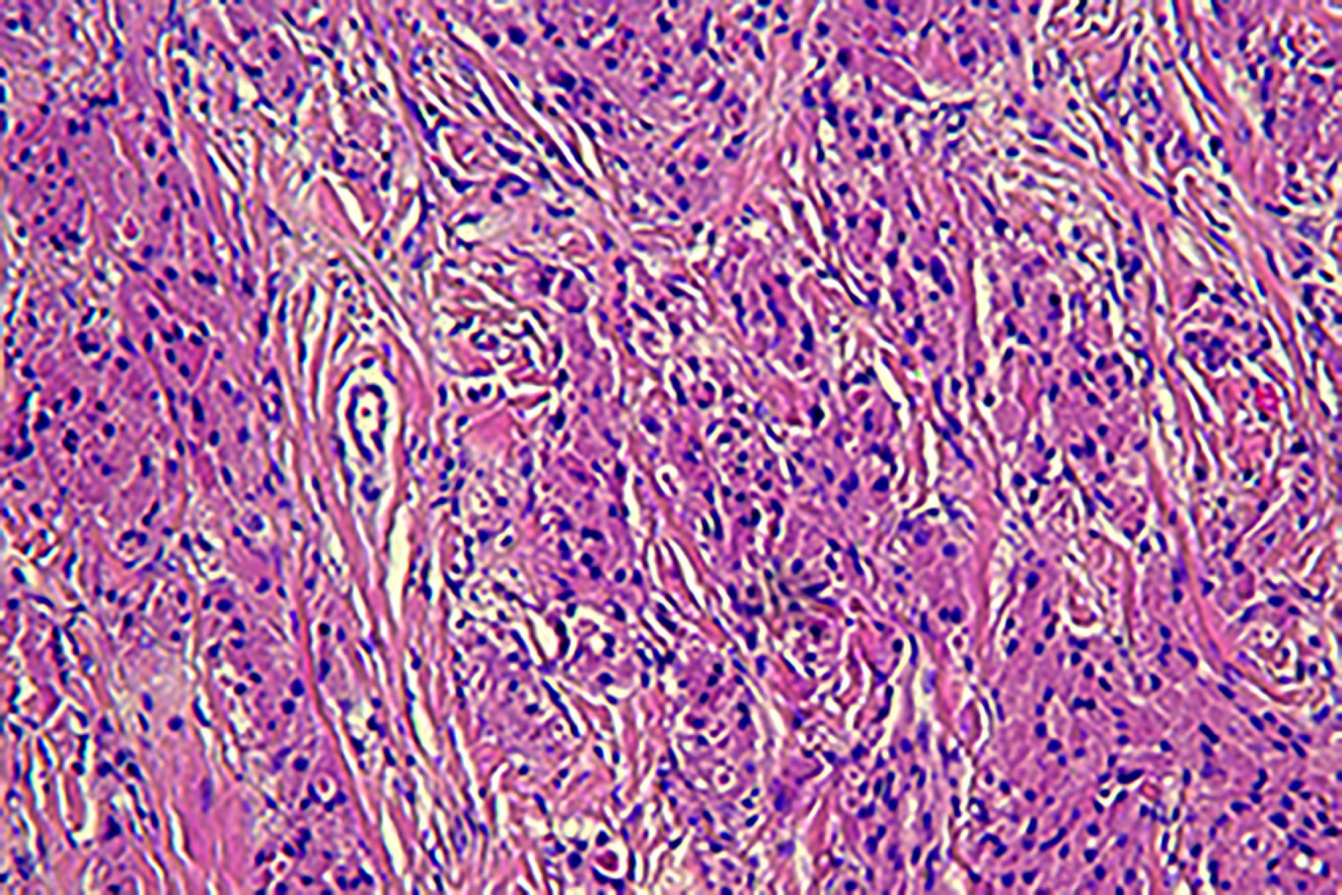
Hematoxylin and eosin staining revealed large cells with cytoplasmic granules.

Pathology: The gross specimen appeared as a white, smooth, shiny, nodular swelling of 2 cm × 1.5 cm, without an envelope, and was grayish-white on section with a medium texture and solid. Light microscopy revealed that the surface was covered with squamous epithelium and some areas of pseudostratified ciliated columnar epithelium, with a tightly packed mass in the submucosa. The tumor cells appeared spindle-shaped, round, polygonal, and irregularly arranged. The tumor cell cytoplasm appeared rich in eosinophilic red cytoplasm and filled with eosinophilic granules. The cell boundaries were indistinct. The nuclei were small, round, or oval, with no chromatin anisotropy, no nuclear fission phase, and obvious nucleoli. The pathological diagnosis indicated GCT. The immunohistochemical findings indicated S-100 (+), NSE (+), CD56 (+), and Ki67 3% (+). The microscopic findings are presented in **Figure 6**.

## Materials and method

The PubMed, EMBASE, and Google Scholar databases were searched with the following search terms: “tracheal granulosa cell tumor”, “granulosa cell tumor”, and “rare airway tumor”. The inclusion criteria were all articles published in PubMed and relevant only to humans. Articles without full texts were excluded. A total of 28 cases of cervical segmental tracheal GCT were identified in this search.

## Results

**Table 1** presents the clinical data of all 29 patients retrieved from the literature. Data were analyzed to elucidate the clinical features of cervical segmental tracheal GCT and assess the efficacy of different treatment modalities. The age range of the patients was 6-64 years, with a mean age of 31.4 years (n=29) and a median age of 31 years. Twenty-five of the 29 patients (86.2%) were female, and 22 had a clear ethnicity, of which 15 (51.7%) were black and three were Asian. Among the 25 patients, 21 (84%) presented with respiratory symptoms, mainly cough, shortness of breath, laryngeal tinnitus, and dyspnea. The mean tumor size at diagnosis was 2.67 cm (n=24), with a long axis range of 0.45-6 cm. Twenty-five cases (86.2%) had a single tumor. Most of the tumors (31) were intracavitary, eight were extracavitary, and five were both intra- and extracavitary. Seven tumors (35%), including the case presented in this study, were documented in the literature as occurring in the posterior tracheal wall. A review of the literature revealed a 62.5% incidence of recurrence or residual tumor in eight patients who underwent endoscopic resection; two of the recurrences were treated by trachelectomy, and two cases were supplemented with endoscopic laser-assisted resection. The incidence of recurrence or residual disease was 50% in the four patients who underwent shave resection and trachelectomy after recurrence in one case. No recurrence was reported in the 12 patients who underwent trachelectomy. One patient underwent electrosurgery and argon plasma coagulation, and two patients underwent transtracheal resection of the mass. Seven of the 18 patients who underwent surgical treatment also underwent thyroidectomy; two cases were found to be cervical tracheal granulosa cell tumors with thyroid carcinoma. A total of 27 patients were treated, of whom one (3.7%) died of postoperative bilateral recurrent laryngeal nerve palsy. The follow-up period ranged from six weeks to nine years, except for two cases without intervention and one death.

**Table 1:** Published data on tracheal granular cell tumors.

Source	Age/ Sex	Race	Complaint	Size (cm)	Solitary/Multiple	Location	Intra/Extraluminal	Results/ Follow-up	Surgical method/ Reconstruction method
Benisch (9)	25/F	Black	Respiratory difficulty asthma	1.5	S	Posterior wall erosion	Intra	NED/15 mo	Tracheal resection and tracheotomy/end to end
Krouse (10)	54/F	Black	NA	2.5	M	Cervical tracheal	Extra	Postmortem finding	None
Thawley (11)	33/F	Black	Painless left neck mass	3.0	S	Left lateral tracheal wall, cricoid cartilage	Extra	Residual disease/8y	Tracheal wall shaving and Thyroid lobectomy/none
Carnalis (12)	45/F	NA	Intermittent hemoptysis	5.5	S	Posterior wall erosion	Intra and extra	NED/3 y	Tracheal resection and tracheotomy/end to end
Polack (13)	29/F	Black	Incidentally found	2.0	S	Anterior wall erosion	Extra	NED/4 wk	Tracheal wall shaving/none
Dunaway (14)	6/F	Black	Intermittent wheezing	2.0	M	Left lateral tracheal wall	Intra	Residual disease/9y	Endoscopic excision
McLain (15)	26/M	Black	Dyspnea	2.0	M	8 cm below the vocal cords	Intra	NED/9 mo	Endoscopic laser excision Tracheal resection and tracheotomy/end to end
Mikaelian (16)	20/F	Black	Cough associated with dyspnea	2.0	S	Posterior wall erosion	Intra	NED/2 y	Endoscopic laser excision Tracheal resection and tracheotomy/end to end
Thaller (17)	31/F	Black	Increasing shortness of breath	6.0	S	Anterior wall erosion	Intra	NED/6 wk	Endoscopic laser excision and tracheotomy
Alessi (18)	33/F	Black	Right upper extremity weakness and dygphngin	NA	S	Low in the neck	Extra	NED/7 y	Partial tracheal resection and thyroid lobectomy/none
Alessi (18)	37/F	Black	Incidentally found	3.0	S	6 cm above the carina,submucogal	Intra	NED/2y	Tracheal resection and tracheotomy/end to end
Solomons (19)	10/M	NA	Acute respiratory distress	NA	S	Posterior tracheal wall and anterior oesophageal wall	Intra	Died postoperatively vocal cords in a paramedian position indicating bilateral recurrent nerve palsies	Partial tracheal resection and tracheotomy/Inferior turbinectomy and the turbinate mucosa sutured in to fill the defect
Oyama (20)	30/F	Asian	Hemoptysis	4.0	S	Posterior tracheal wall	Intra	NED/1.5y	Tracheal resection and tracheotomy/end to end
Burton (21)	14/F	Black	NA	NA	M	Cervical tracheal	Intra	NED/4 mo	Tracheal resection/end to end
Burton (21)	37/F	Black	A chronic cough and upper respiratory infection	4.0	S	Right lateral tracheal wall erosion	Extra	NED/1 y	Tracheal shaving and partial tracheal resection Thyroid lobectomy
Burton (21)	19/F	NA	Intractable asthma	1.5	S	Cervical tracheal	Intra	NED/8 y	Endoscopic excision,
Burton (21)	43/F	Black	Severe asthma and new-onset dysphagia	4.0	S	Anterior wall erosion	Intra and extra	NED/6 mo	Tracheal resection and tracheotomy/end to end
Spandow (22)	12/M	White	Increasing difliculty in breathing	2.0	S	Left lateral tracheal wall erosion	Intra and extra	NED/18 mo	Partial tracheal resection and thyroid lobectomy and tracheotomy/end to end
Thomas (23)	46/F	NA	Dyspnea	4.5	S	Upper part of tracheal	Intra	Residual disease/9y	Successive endoscopic excision, laser
Frenckner (24)	28/F	White	NA	NA	S	Cervical	Intra	NED/3 y	Transtracheal enucleation
Desai (25)	10/F	NA	Obstructive sleep apnea and exercise-induced asthma	NA	S	Posterior tracheal wall	Intra	NED/10 y	Tracheal fissure and excision and tracheotomy/
Kintanar (26)	35/F	NA	Painless right neck mass	2.2	S	Right lateral tracheal wall	Extra	NA	Tracheal shaving/Thyroid lobectomy
Daniel M (27)	29/F	Black	Dyspnea	3.0	S	Right lateral tracheal wall erosion	Intra and extra	NA	None
Ipakchi (8)	22/F	Black	Shortness of breath, expiratory stridor	3.0	S	Right lateral tracheal wall	Intra	NED/18 mo	Endoscopic resection
Colella (28)	58/M	White	trachyphonia and occasionally dysphonia Painless right neck mass	1.1	S	Right lateral tracheal wall erosion	Extra	NED/30 mo	Thyroidectomy, partial tracheal resection
Lee (29)	45/F	Asian	Cough,shortness of breath	3.0	S	Anterior tracheal wall	Intra	NED/13 mo	Endoscopic resection, thyroid lobectomy
Guarnieri (6)	64/F	White	Dyspnoea	1.3	S	Left lateral tracheal wall	Intra	NA	Electro surgery and argon plasma coagulation
Fama (30)	26/F	NA	Painless right neck mass	1.9	S	Right lateral tracheal wall	Extra	NED/12 mo	Tracheal shaving/Thyroid lobectomy
Currentstudy	45/F	Asian	Activities difliculty in breathing	2.0	S	Posterior wall erosion	Intra and extra	NED/3 mo	Partial posterior wall resection Reconstruction tracheal by two lateral edges of the fascia tissue flap

NA, not available; F, female; M, male; S, solitary; M, multiple; Intra, intraluminal; Extra, extraluminal; NED, no evidence of disease; DWD, died with the disease.

## Discussion

GCT was discovered and first described in 1926 after a long case study. It is now known to originate from neuronal tissue, histiocytes, or Schwann cells. GCT can occur anywhere in the body, usually involving the skin, breast, and especially the gastrointestinal tract (e.g., mouth, tongue, and esophagus); however, GCT in the respiratory tract, especially in the trachea and bronchi, is rare (32). The majority of GCTs reported in the literature are benign, with only 1−2% being malignant, specifically in cervical tracheal GCTs.

The peak age of onset of GCT is between 30 and 50 years, with a higher prevalence in women and patients of black ethnicity. The observation that most GCT cases occur in females may support a correlation between hormones and disease (33), although there is a lack of data to establish a clear correlation. However, 86.2% of the patient data we retrieved were from females, and the cervical segment tracheal GCTs were consistent with this profile.

History and physical examination provide important clues to diagnosing GCT of the cervical trachea. A tumor located outside the cavity may appear as a painless mass in the neck. Therefore, GCT of the trachea is often misdiagnosed as bronchial asthma (34), chronic bronchitis, and tumors of thyroid origin. The differential diagnosis should include subsonic masses, benign and malignant tumors of the trachea, and esophageal and thyroid tumors. Specifically, two cases of thyroid carcinoma with cervical tracheal granulosa cell tumor (28, 30) were identified; these could easily lead to misdiagnosis and increase the likelihood of adverse events during the treatment course. Hence, examination methods and the capability to differentiate and diagnose diseases are particularly important. If a tumor arises intraluminally, endoscopy is the mode for visualization of tumor location, size, and intraluminal status and can be used to define the pathology by tissue biopsy. Tomographic CT scans of the trachea and larynx, both of which are the primary methods of examining tracheal GCT, can determine the location and extent of tumor infiltration. If a tumor occurs adjacent to the thyroid gland outside the lumen, pathological examination and CT of ultrasonic guided neoplasm needle biopsy are key to the differential diagnosis.

Pathological examination is the gold standard for confirming the diagnosis of GCT, which typically shows loosely arranged polygonal cells of medium or large size. The cytoplasm is rich in eosinophilic granules, with rare nuclei and nucleoli. Immunohistochemistry provides an important diagnostic aid for pathological examination, and the expression of S-100 protein and neuron-specific enolase (NSE) can be important evidence for diagnosis (35).

The treatment modality for cervical segmental tracheal GCT should be selected according to the site of the tumor and the degree of infiltration. An analysis of the treatment outcomes of the retrieved cases showed a 62.5% incidence of recurrence or tumor residuals in endoscopic laser-assisted resection of intraluminal tumors, with no deaths, and a 50% incidence of recurrence or tumor residuals in shave resection of extraluminal tumors. Among the cases in which both procedures were used, three had tumor residuals, and although the progression-free survival period was between eight and nine years, there are currently insufficient data to suggest that this inert biological behavior of GCT is common. In intraluminal or extraluminal tumors without trachelectomy, enhanced management of the tumor base may be one way to reduce the recurrence rate, but again, data to support this are insufficient. Cervical trachelectomy has the highest cure rate with a mortality rate of 4%, while the cause of death in the case presented here was bilateral recurrent laryngeal nerve palsy due to intraoperative manipulation. The literature shows that trachelectomy has a high cure rate and a lower mortality rate than previously reported and is a reasonable approach for both radicality and safety; however, this procedure often requires tracheotomy, which is a factor affecting the patient’s postoperative quality of life. Radical surgery usually needs to include thyroid lobectomy, depending on the extension and involvement of the tumor in the paratracheal region. To date, available data about laser resection are similarly sparse. Owing to limited data, a reasonable approach could be based on endoscopic therapy as the preferred treatment, and trachelectomy should be reserved for larger lesions that prevent disease recurrence or threaten airway patency (36). Because of the rarity of the disease, data on recurrence rates are lacking, and follow-up plans cannot be defined.

A literature survey yielded six cases with tumors in the cervical segment of the trachea. Four cases (66.6%) underwent tracheal sleeve resection with end-to-end anastomosis, and one underwent partial tracheal resection with the application of inferior turbinate mucosa to repair the tracheal membrane. Tracheotomy was performed in all six patients, and a tracheal tube was left in place. In the case presented in this study, we attempted to remove the membranous tumor while preserving the intact tracheal cartilage ring to avoid tracheotomy when it was clear that the mass did not invade the tracheal wall or the anterior esophageal wall. During the operation, we used the intertracheoesophageal approach to expose the tumor by freeing the lateral side of the thyroid gland; the procedure could retain the complete blood supply to the thyroid gland without affecting the function of the parathyroid gland and keep the recurrent laryngeal nerve above the tumor during the resection. Studies have shown that fascial tissues are hypometabolic, easily survivable, and resistant to saliva and infection, providing an excellent seal and structural stability for respiratory epithelialization (31). Furthermore, several reports on the successful repair of the residual larynx with banded myofascial flaps during laryngeal cancer surgery support the high survival rate of banded myofascial flaps (29). Therefore, we attempted to reconstruct the tracheal membrane after tumor resection by applying an adjacent double-tipped banded myofascial tissue flap. To prevent postoperative asphyxia, we created a 1-cm-long tracheal skin fistula without a tracheal tube, and the tracheal skin fistula of the patient was always sealed and facilitated oral and nasal breathing after surgery. We believe that the advantages of the banded myofascial flap for tracheal membrane repair include no additional incision, proximity to the recipient site, good blood supply in the form of an upper and lower double-tip, no twisting of the double-tip during suturing, moderate fascial tension, and avoidance of tracheotomy, making it an ideal graft for reconstruction of cervical tracheal membrane defects. However, the limitations of using banded myofascial flaps to repair tracheal defects, including the maximum distance between the double-tipped banded myofascial flap and the membranous defect, and the survival rate of single-tipped fascial flaps, remain unknown and need to be verified and promoted by more data in the future.

## Conclusions

GCT of the cervical segment trachea is a rare disease with a lack of specific information on its clinical presentation and imaging, and histopathological examination is the gold standard for diagnosis. Furthermore, while electronic bronchography is an important diagnostic method, it has limitations in terms of treatment. GCT trachelectomy is the best treatment for large infiltrating cervical tracheal tumors. Finally, in GCT of the cervical trachea, preservation of the tracheal cartilaginous ring and membrane reconstruction with a double-tipped banded myofascial flap is a feasible approach.

## Data availability statement

The original contributions presented in the study are included in the article/supplementary material. Further inquiries can be directed to the corresponding author.

## Ethics statement

Written informed consent was obtained from the individual(s) for the publication of any potentially identifiable images or data included in this article.

## Author contributions

All authors contributed to the drawing up and correction of the paper. All authors contributed to the article and approved the submitted version.
